# Somatic Mutations in Core Spliceosome Components Promote Tumorigenesis and Generate an Exploitable Vulnerability in Human Cancer

**DOI:** 10.3390/cancers14071827

**Published:** 2022-04-04

**Authors:** Claudio Sette, Maria Paola Paronetto

**Affiliations:** 1 Department of Neuroscience, Section of Human Anatomy, Catholic University of the Sacred Heart, 00168 Rome, Italy; claudio.sette@unicatt.it; 2 GSTEP-Organoids Core Facility, Fondazione Policlinico Agostino Gemelli IRCCS, 00168 Rome, Italy; 3 Department of Movement, Human and Health Sciences, University of Rome “Foro Italico”, Piazza Lauro De Bosis, 6, 00135 Rome, Italy; 4 Laboratory of Molecular and Cellular Neurobiology, Fondazione Santa Lucia, IRCCS, Via del Fosso di Fiorano 64, 00143 Rome, Italy

**Keywords:** spliceosome, cancer, somatic mutations, alternative splicing

## Abstract

**Simple Summary:**

High throughput exome sequencing approaches have disclosed recurrent cancer-associated mutations in spliceosomal components, which drive aberrant pre-mRNA processing events and support the tumor phenotype. At the same time, mutations in spliceosome genes and aberrant splicing regulation establish a selective vulnerability of cancer cells to splicing-targeting approaches, which could be exploited therapeutically. It is conceivable that a better understanding of the mechanisms and roles of abnormal splicing in tumor metabolism will facilitate the development of a novel generation of tumor-targeting drugs. In this review, we describe recent advances in the elucidation of the biological impact and biochemical effects of somatic mutations in core spliceosome components on splicing choices and their associated targetable vulnerabilities.

**Abstract:**

Alternative pre-mRNA processing enables the production of distinct mRNA and protein isoforms from a single gene, thus greatly expanding the coding potential of eukaryotic genomes and fine-tuning gene expression programs. Splicing is carried out by the spliceosome, a complex molecular machinery which assembles step-wise on mRNA precursors in the nucleus of eukaryotic cells. In the last decade, exome sequencing technologies have allowed the identification of point mutations in genes encoding splicing factors as a recurrent hallmark of human cancers, with higher incidence in hematological malignancies. These mutations lead to production of splicing factors that reduce the fidelity of the splicing process and yield splicing variants that are often advantageous for cancer cells. However, at the same time, these mutations increase the sensitivity of transformed cells to splicing inhibitors, thus offering a therapeutic opportunity for novel targeted strategies. Herein, we review the recent literature documenting cancer-associated mutations in components of the early spliceosome complex and discuss novel therapeutic strategies based on small-molecule spliceosome inhibitors that exhibit strong anti-tumor effects, particularly against cancer cells harboring mutations in spliceosomal components.

## 1. Introduction

Pre-mRNA splicing is catalyzed by the spliceosome, a dynamic ribonucleoprotein (RNP) enzyme that assembles step-wise on each intron to be spliced [1]. During the splicing cycle, a dynamic network of RNA–RNA and protein–RNA interactions occurs [1,2], which is essential for the formation, rearrangement, and dissociation of the spliceosomal complexes from the nascent transcript [3]. In the first step of assembly of the spliceosome, named complex E, U1 snRNP, SF1, U2AF65, and U2AF35 bind to the splicing cis-elements at the 5′ splice site (ss), the branch point sequence (BPS), the polypyrimidine tract (PPT), and the acceptor site, respectively [4]. In yeast, the BPS is well conserved [5], whereas in humans it is highly degenerated [6]. The BPS in human transcripts is usually recognized along with the downstream PPT, where U2AF65 binds and forms a heterodimer with U2AF35 [7], possibly binding to the invariant AG dinucleotide at the 3′ ss [8]. The presence of uridines over cytidines in the PPT is preferred for a stronger recognition [9].

**Figure 1:**
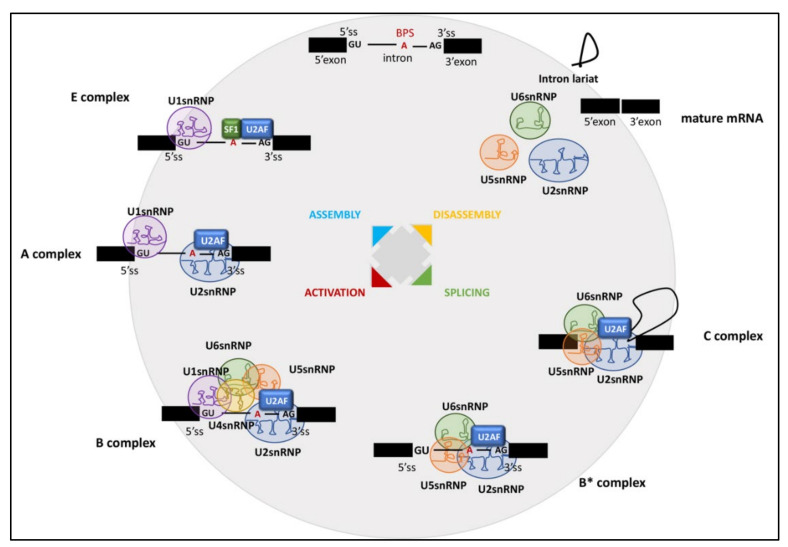
Pre-mRNA assembly by the spliceosome machine. The stepwise interaction of the spliceosomal snRNPs and RBPs in the removal of an intron from a pre-mRNA containing two exons (black).

In the transition from the E to A spliceosome complex, U2AF65 and U2AF35 binding facilitate the substitution of SF1 with U2snRNP at the BPS [1] (Figure 1). Notably, introns carrying a long stretch of PPT do not require U2AF35 for this substitution, which is called “AG-independent” 3′ splice site [10,11]. Conversely, introns with a short or degenerated PPT require both U2AF65 and U2AF35 for this substitution (“AG-dependent” 3′ splice site) [10,11,12]. Subsequently, the U4/U6/U5 tri-snRNP is integrated into the spliceosome to form the complex B, and the initial assembly of the spliceosome is completed [1]. Notably, the invariant AG dinucleotides, BPS and PPT, are frequently reported as targets of mutations that cause human diseases by generally leading to skipping of one or more exons in transcript of disease-related genes [6,13,14].

The B complex is then activated with the release of the U1 and U4 snRNPs, yielding the B-act complex [1]. Thereafter, the ATP-dependent DEAH-box protein PRP2 gives rise to a structural rearrangement leading to the catalytically activated spliceosome (B* complex) [15], which is then converted into the C complex (Figure 1). During this first catalytic step, the pre-mRNA is cleaved at the 5′ splice site forming the lariat structure. The second catalytic step is promoted by the ATP-dependent DEAH-box protein PRP16 [16] leading to ligation of the two exons. After release of the spliced intron, the spliceosome dissociates, and the snRNPs are recycled for additional rounds of splicing [1]. To achieve the removal of introns and joining of exons, breakage and formation of phosphodiester bonds is employed in two successive transesterification reactions [1] (Figure 1). In addition to the core spliceosome, accessory regulatory RNA-binding proteins (RBPs) can affect these splicing reactions. RBPs act as activators or repressors of splicing by binding to exonic and intronic enhancer or silencer elements [17]. 

Large-scale genome sequencing analyses led to the identification of recurrent somatic mutations in components of the spliceosome and in genes encoding RBPs in several human malignancies, providing genetic evidence of a direct link between splicing misregulation and cancer pathogenesis. Mutations in spliceosomal genes have been reported in myelodysplastic syndromes [18], chronic lymphocytic leukemia [19], acute myeloid leukemia [20], breast cancer [19,21], lung adenocarcinoma [22,23], and uveal melanoma [24]. Interestingly, most of the identified mutations affect components of the early spliceosome complexes E and A, whose deregulation strongly impacts the assembly of the machinery and, as a consequence, splicing decisions.

In this review, we will discuss splicing alterations detected in human tumors. In particular, we will focus on mutations occurring in components of the E complex. This complex is the earliest spliceosome precursor, which is responsible for definition of the actionable 5′ss and BPS and, therefore, for the initial steps of the splicing process. The identified mutations can affect both RNA–RNA and protein–RNA interactions. Remarkably, these mutations exhibit cell-type specificity, thus displaying defined roles in the development and progression of specific tumor subtypes. In this way, spliceosome mutations not only contribute to cell transformation, but also define and shape human tumors.

## 2. U2AF1 Mutations in Human Malignancies

**Figure 2:**
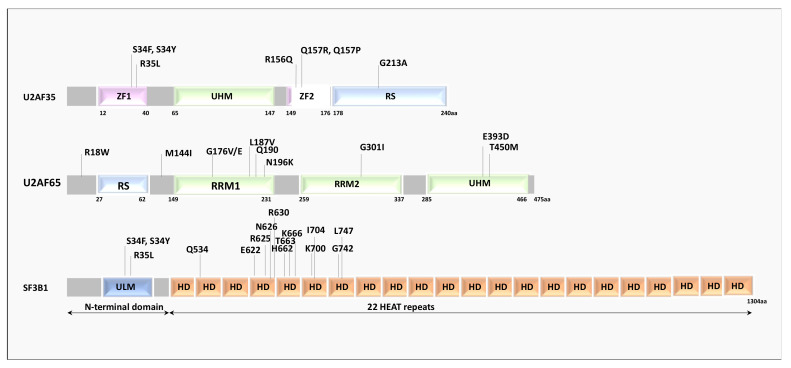
U2AF35, U2AF65, and SF3B1 mutations in an individual with hematological malignancies. Schematic representation of U2AF35, U2AF65, and SF3B1 protein domains and recurrent somatic mutations identified in cancer patients within each domain. ZF, zinc finger; UHM, U2AF homology motif; RS, Arginine Serine domain; RRM, RNA-recognition motif; ULM, U2AF ligand motif; HD, HEAT (Huntingtin, Elongation factor 3, protein phosphatase 2A, Targets of rapamycin 1) Domain.

The *U2AF1* (U2 small nuclear RNA auxiliary factor 1) gene encodes a small (~35 kDa) non-snRNP protein involved in the early phases of splicing regulation. U2AF35 protein structure is highly conserved across species and comprises two zinc-finger (ZnF) domains, an RNA-recognition motif (RRM), and a C-terminal arginine/serine-rich-domain [25,26]. During the splicing cycle, U2AF35 binds to the AG nucleotide at the 3′ splice site of regulated exons and assists U2AF2 (U2AF65) in the recruitment of U2 snRNP, thus stabilizing the base pairing to the BPS. In this way, it ensures proper assembly of the E/A complex [1]. Mutations in the *U2AF1* gene have been detected in several hematological malignancies, including myelodysplastic syndromes (MDS) [18,27], chronic lymphocytic leukemia (CLL) [28], acute myeloid leukemia (AML) [29], and, in solid tumors, including breast cancer [30], lung adenocarcinoma [23], as well as in other malignancies, albeit at lower rates. Interestingly, the identified mutations cluster in two hotspots located within the two zinc-finger domains (Figure 2) [18] and primarily affecting two amino acid residues, Serine 34 (S34) and Glutamine 157 (Q157).

Recombinant expression of mutant U2AF1^S34F/Y^ mutants in HeLa cells induces global splicing abnormalities, preferentially impacting exon cassettes and alternative 3′ splice sites [22]. At the molecular level, the S34F mutant promotes splicing of exons with a C or A preceding the AG dinucleotide of the 3′ splice site and to repress those exons exhibiting a U at this position [20,22]. Furthermore, *U2AF1^S34F^* inducible expression in mice results in phenotypes associated with MDS, including leukopenia, increased apoptosis of maturing cells in the bone marrow, and progenitor cell expansion [31]. Although these mice do not develop MDS or AML, they express defective splicing of genes recurrently mutated in MDS/AML, such as *GNAS*, *PICALM, H2AFY, BCOR, KDM6A, KMT2D (MLL2),* and *MED24,* thus likely contributing to the altered hematopoiesis that is characteristic of MDS patients [31].

In addition to aberrant splicing, U2AF35^S34F^-transformed cells show altered mRNA 3′ end processing, resulting from increased use of a distal cleavage and polyadenylation sites [32]. Among the affected targets, the autophagy-related factor 7 (Atg7), which encodes an essential autophagy factor, was directly linked to the neoplastic phenotype. Indeed, U2AF35^S34F^-dependent distal cleavage and polyadenylation in *Atg7* mRNA causes defects in autophagy and leads to mitochondrial dysfunction [32]. This failure results in genomic instability that predisposes cells to secondary oncogenic mutations, ultimately contributing to transformation [32].

While several studies analyzed the more frequent S34F/Y mutations, only a few have investigated the molecular consequences of the Q157R/P alleles. Interestingly, in addition to causing the Q157R substitution in the U2AF35 protein, the c.470A>G mutation also generates an alternative 5′ splice site that leads to the deletion of four amino acids within the second ZnF domain (Q157Rdel) [33]. This deletion does not involve cysteine and histidine residues complexing with the zinc, and the structure of the mutated ZnF domain should retain its functionality. However, the loss of a short helix could slightly alter the orientation and distance between the Zn complexing residues. As a result, the Q157Rdel mutant shows compromised RNA binding specificity and, consequently, altered splicing specificity [33]. Likewise, the cancer-associated Q157R and Q157P point mutants also target distinct splice sites, suggesting differential RNA-binding preferences [33].

The heterozygous mutations in *U2AF1* gene were also reported to increase the formation of RNA:DNA hybrid adducts, named R-loops [34], which can generate DNA damage if they are not properly resolved. In line with this effect, expression of the mutant U2AF35 proteins triggered activation of the ataxia telangiectasia and Rad3-related protein (ATR) pathway, thus causing elevated γ-H2AX signals and cell cycle arrest due to DNA replication stress [34]. Importantly, overexpression of RNase H was sufficient to partially suppress R-loop formation and to rescue the proliferation defect of hematopoietic cells harboring these mutations [34]. These findings suggested that inhibition of the ATR pathway may further impair survival of these cells, opening the path to novel therapeutic opportunities in hematologic malignancies harboring *U2AF1* mutations.

Remarkably, conditional *U2af1* knockout mouse in hematopoietic stem cells and progenitor cells (HSPC) results in pancytopenia, dramatic decrease in bone marrow cellularity, defects in HSC repopulation capacity, and increased cell death in hematopoietic progenitors and early lethality in mice [35]. *U2af1* deficiency also induces splicing alterations and perturbed expression of genes important for HSPC function, including the NFYA and PBX1 transcription factors [35]. These findings suggest an important role for U2AF35 in the physiological maintenance and function of HSPC during hematopoiesis and highlight potential downstream effectors of the *U2AF1*-mutant phenotype which might represent therapeutic targets for hematologic malignancies.

## 3. *U2AF2* Mutations in Human Malignancies

Albeit at lower frequency, cancer-related mutations were also identified in the *U2AF2* gene, encoding the U2AF larger subunit U2AF65. U2AF65 protein contains two central RRMs (RRM1 and RRM2) that recognize the PPT, a C-terminal U2AF2 homology motif (UHM), which recognizes the BPS in complex with SF1, and the N-terminus, which interacts with U2AF35 and allows its correct recognition of the AG dinucleotide at the 3′ splice site [36]. After U2AF35 binding, U2AF65 recruits SF3B1 to the assembling spliceosome [36].

In most cases, *U2AF2* mutations affect residues located in several domains of the protein, such as the RRM1 and RRM2, the N-terminal region for heterodimerization with U2AF35, and the C-terminal protein-interaction motif. X-ray crystallography and NMR data have established that the two RRMs recognize a continuous nine-uridine PPT in an “open” configuration [37,38], whereas in the absence of RNA, a “closed” U2AF65 conformation occurs, in which the RNA-binding surface of RRM1 is masked by the RRM2, stabilized in the heterodimer with the U2AF35 subunit [39]. Cancer-associated *U2AF2* mutations mostly fall within the RRM1 motif, at the interface that interacts with nucleotides in the 3′ half of the PPT. The Asparagine 196 (N196) residue, which is mutated in Lysine in some AML patients, is involved in uridine binding [40]; the Glutamine 190 (Q190) residue, mutated to leucine in some CLL patients, is involved in the binding to the terminal nucleotide of the PPT [40], which is also recognized by the Aspartate 231 (D231), mutated to asparagine in some solid cancers [40]. The Glycine 176 (G176V/E) and Glutamine 190 (Q190L) mutations were found in lung adenocarcinoma and in liver, breast, and colon cancers [40]; the Leucine 187 to Valine (L187V) amino acid change, also residing in the RRM1, was observed in six patient samples with hematologic malignancies, including AML, CMML, and MDS [40]. On the other hand, mutations at the RNA interface of RRM2 drive U2AF65–RNA binding [40,41,42]. For instance, the Glycine 301 to aspartate mutation in the RRM2 was found in patients with colorectal or prostate carcinomas or to serine in a papillary renal cell carcinoma [40]. The G301I mutation could reduce U2AF2–PPT binding affinity [40,42]. Collectively, *U2AF2* cancer-associated mutations falling within the two RRMs impact the equilibrium between the “closed” (unbound) and “open” (RNA binding-competent) conformations, alter binding of the protein to the PPT, and dysregulate pre-mRNA splicing, similarly to U2AF1 mutations. 

Outside of the RRMs, two recurrent mutations were also identified in the UHM domain, Threonine 450 (T450M), and Glutamate 393 (E393D). These mutations affect the interaction of U2AF65 with SF1 [40]. Future studies are needed to unravel the potential roles of recurrent U2AF2 mutations in the transformation of normal cells to cancers and to elucidate their genome-wide effects of splicing.

## 4. *SF3B1* Mutations in Human Malignancies

The SF3b complex is a component of the U2 small nuclear ribonucleoprotein (snRNP), which plays key roles in recognizing the BPS and facilitates spliceosome assembly and activation (Figure 1) [1]. Seven SF3b components have been identified in yeast and human [43]. SF3B1 (Splicing Factor 3b Subunit 1) is the largest protein in the complex and mediates U2 snRNP recruitment to the BPS by interacting with the intronic region of the pre-mRNA. SF3B1 contains several domains, including a stretch of U2AF ligand motifs (ULMs) at its N-terminus, which can specifically interact with the U2AF homology motif (UHM) of U2AF65, and the HEAT (Huntingtin, Elongation factor 3, protein phosphatase 2A, Targets of rapamycin 1) domain, formed by 22-tandem-repeats forming a superhelix (Figure 2). Base pairing between the U2 snRNP and the BPS is essential for pre-mRNA splicing. As part of the SF3b complex, SF3B1 associates with the SF3a complex and U2 snRNP to form the 17S U2 complex [44]. By anchoring the U2 snRNP to the pre-mRNA, this complex allows correct positioning of the branch-point adenosine for the nucleophilic attack from the 5′ splice site [44]. U2 snRNP binds to BPSs via SF3B14 and to U2AF2 via SF3B1, thus leading to the formation of the spliceosomal A complex [1].

SF3B1 represents the most frequently mutated component of the spliceosome in cancer. SF3B1 mutations were identified in a variety of myeloid malignancies, with high recurrence in MDS subtypes and in CLLs [18]. Notably, SF3B1 mutations are clustered in several hot spots localized in regions that are critically involved in the tertiary structure of the protein [45]. Most of these substitutions induce changes in the conformation and curvature of the HEAT superhelix [45], thus diminishing the interaction of SF3B1 with the pre-mRNA and other spliceosomal proteins, including p14 and U2AF65, and leading to the selection of alternative BPS sequences [46]. In particular, the R625, N626, R630, T663, and K666 residues are involved in intramolecular hydrogen bonds; the I704, G742, and L747 are tightly packed in hydrophobic interfaces and their substitutions likely result in conformational clashing; the Q534, A744, and A745 residues are located in α helices, and their mutations can lead to structural collapse [45].

The hot spot residue K700, together with other recurrently mutated residues within the H3–H4 helices of the HEAT domain, fall in the immediate vicinity of the basic surface interacting with the UHM domain of U2AF65 [45], suggesting a role in BPS recognition or interaction with other spliceosomal proteins. In support of this hypothesis, it was shown that the SF3B1 K700E mutant promotes usage of a different BPS compared to wild type SF3B1 and recognizes cryptic 3′ splice sites with short and weak PPT located upstream of the canonical 3′ splice site [47]. Similarly, the R625 and K666 mutations result in deregulated splicing at a subset of splice sites, mostly involving the usage of alternative or cryptic 3′ splice sites characterized by an enrichment of adenosines and a short PPT [19,46]. These splicing abnormalities have also been reported in CLL samples and solid tumors displaying SF3B1 mutations, including breast carcinoma and melanoma [47]. However, protein–protein crosslinking and in vitro binding assays showed that at least the K700E mutation does not affect the stability of the SF3b–U2AF65 interaction nor RNA binding [45], suggesting that other spliceosomal components are involved in this deregulation.

SF3B1 mutations in the K700 and K666 residues have been reported also in breast tumors [21,48] and in pancreatic ductal adenocarcinomas [49], whereas mutations of residues R625 and K666 were found in uveal melanomas [24,50,51] and cutaneous melanomas [52,53]. In breast tumors, SF3B1 mutations were significantly associated with ER-positive disease, often displaying concurrent AKT1 genomic alterations [21]. However, the implications of these association are currently unknown.

Heterozygous, hematopoietic-restricted expression of *SF3B1^K700E^* is sufficient to cause MDS features in mice, including a macrocytic anemia due to a block in terminal erythropoiesis, erythroid dysplasia, and expansion of LT-HSCs in the bone marrow [54]. Interestingly, more than 30% of murine and human aberrantly spliced genes in SF3B1 mutated context undergo NMD [54], suggesting a widespread impact on the functionality of the transcriptome.

These findings point to SF3B1 as the most commonly mutated spliceosomal component in cancer and suggest that this protein can represent a valuable target for novel therapeutic strategies.

## 5. Splicing-Based Therapeutic Strategies

Targeting splicing is becoming a reliable tool to counteract malignant transformation and cancer progression. Small-molecule modulators of the spliceosome have demonstrated anti-tumor effects and are particularly active against cancer cells harboring mutations in spliceosomal components [55]. It is likely that tumor cells can exploit lower splicing fidelity to their advantage, but further deficiency in the process is not tolerated. In support of this notion, all mutations in spliceosome genes are found in heterozygosity in tumor cells, and they are mutually exclusive [18,56]. Thus, a certain level of splicing dysregulation may allow evolution of tumor cells by amplifying the repertoire of splice variants and/or by inactivating the expression of tumor suppressor genes. However, this defective regulation exposes them to a vulnerability, as they become more sensitive to further splicing inhibition. Interestingly, higher sensitivity to splicing inhibition was also observed in MYC-driven cancers [57]. In that scenario, the higher transcriptional rate caused by overexpression of the oncogenic MYC transcription factor generates a burden on the splicing machinery, which can be exploited therapeutically [57]. These observations have raised considerable interest on splicing regulation in health and disease [58] and have fostered studies aimed at evaluating the potential of splicing inhibitors as anticancer agents [59,60].

The field of splicing inhibition as anticancer treatment has sparked since the discovery of Yoshida and colleagues in 2007 which showed that the anti-proliferative properties of the natural product FR901464 and of its methylated derivative, spliceostatin A, were related to their ability to bind and inhibit SF3B1 [61]. Since then, several other compounds that modulate pre-mRNA processing have been identified, including pladienolide B and its derivatives E7107 and H3B-8800 [59,62]. As mentioned above, malignancies displaying mutations in spliceosomal genes are particularly sensitive to spliceosome inhibitors, thus offering additional therapeutic opportunities. For example, HSPCs expressing the SF3B1^K700E^ mutant showed increased sensitivity to the spliceosome modulator E7107, both in vitro and in vivo [54]. The orally available SF3B inhibitor H3B-8800 showed even higher selectivity for both hematological and solid tumor cells with respect to normal cells [63]. These studies have raised the hope that targeting the splicing machinery in cancer may also be feasible and safe in a clinical setting. Overall, *SF3B1* or other spliceosome mutations may open a therapeutic window for the use of spliceosome modulators in the treatment of malignancies, opening the path to personalized treatments.

In the following paragraphs, we will describe the most characterized splicing inhibitors and discuss their potential as novel therapeutic treatments for human malignancies, including ongoing clinical trials (Table 1).

### 5.1. FR901464, Spliceostatin A, and Meayamycin

**Figure 3:**
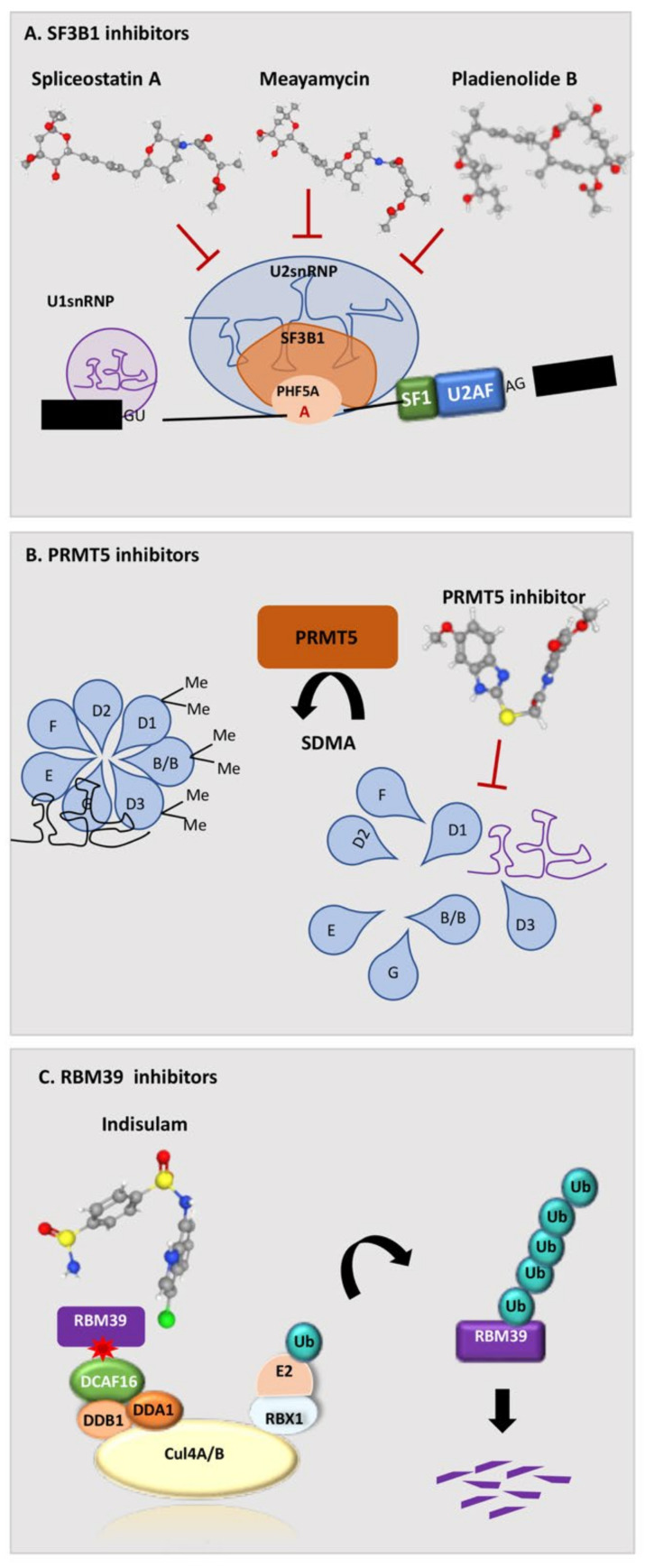
Splicing-based therapeutic strategies in cancer. (**A**) SF3B1 binding agents, including spliceostatin A, meayamycin, and Pladienolide B, interact with SF3B1, thus blocking its binding to the branch point. (**B**) PRMT5 inhibitors inhibit PRMT5-mediated symmetric demethylation of arginines’ (SDMA) on Sm (D1, B/B, D3) proteins, which is required for spliceosome assembly. (**C**) Indisulam links the E2 ubiquitin ligase complex to RBM39 through the adaptor DCAF15, thus leading to polyubiquitination of RBM39 and its proteasome-mediated degradation.

FR901464 was isolated in 1996 from the culture broth of *Pseudomonas* No Sp2663 [64] This compound exhibited potent anti-tumor activities against murine and human tumor cell lines in vitro. Subsequently, it was shown that the natural product FR901464 and its methylated derivative, named spliceostatin A (SSA), were able to inhibit in vitro splicing by directly binding to the SF3b complex and promoting pre-mRNA accumulation [61]. Importantly, cell treatment with these compounds resulted in leakage of pre-mRNA to the cytoplasm, thereby allowing translation of unspliced mRNAs and reproducing the effect exerted by SF3b knockdown [61]. The nuclear-retained pre-mRNAs were significantly longer in size than leaked pre-mRNAs in cells treated with SSA [82], suggesting that short pre-mRNAs can escape the nuclear retention more efficiently. However, another possibility is that the reduced abundance of long pre-mRNAs in the cytoplasm is due to rapid degradation by nonsense mediated mRNA decay (NMD), because long pre-mRNAs retaining introns in the coding sequence are more likely to harbor pre-termination codons (PTCs) that trigger NMD. Mechanistically, SSA was shown to alter the interaction of SF3b with the pre-mRNA, and 3′ splice sites were shown sequence-related different sensitivity to SSA treatment (Figure 3) [61,62]. Indeed, SSA favored specific alternative splicing changes without causing a global splicing inhibition [61]. Since several splicing changes affected genes related to cell cycle control, this study also provided a direct link between the phenotypic effects of the drug and its molecular mechanism. It also suggested that SF3b activity prevents the occurrence of nonproductive base-pairing interactions of the spliceosome with the pre-mRNA, thus acting as an important checkpoint for the recognition of the functional splice sites. 

The FR901464 analogue meayamycin was identified in 2009 and showed higher antiproliferative activity against human cancer cells than previous compounds [69]. Moreover, meayamycin was more active toward lung cancer cells with respect to non-tumoral cells [69] and, when administered to healthy bone marrow cells, it induced phenotypic features observed in tumoral hematological cells [83]. Similar properties were also shown for sudemycins, other FR901464 derivatives that are structurally less complex and not degraded by human plasma [65]. Sudemycin E binds to SF3B1 and induces dissociation of the U2 snRNPs [65]. Genome-wide analyses of the transcriptome showed a rapid switch in alternative pre-mRNA splicing upon treatment with sudemycin E. The splicing inhibition was followed by changes in overall gene expression and arrest in the G2 phase of the cell cycle, which correlated with loss of the H3K36me3 modification in the chromatin encoding the regulated exons [70]. These findings suggested that, in addition to altering splicing, sudemycin E can impact active chromatin inducing local chromatin condensation [70]. Another similar compound, sudemycin D6, exhibited higher antiproliferative activity toward haematopoietic cells expressing mutant U2AF1(S34F), including cells isolated from patients [84]. Interestingly, although structurally similar, SSA and Sudemycins differentially affected splicing of target genes. Both FR901464 analogues act at the latest stages of intron selection by the U2 snRNP by “locking” the SF3B1 subunit in an open conformation and preventing the formation of the extended U2/intron duplex [85]. However, sudemycins preferentially regulate exon skipping events, whereas SSA displays stronger effects on intron retention [62]. The differential response of splicing outcome to these drugs in live cells was related to specific sequence features of the regulated introns and exons, including their length and the strength of the BPS and PPT [62]. These observations suggest that combined treatments with different spliceosome inhibitors may elicit more efficacious anticancer effects by targeting different splicing events.

### 5.2. Pladienolide and its Derivatives E7107, H3B-8800, and FD-895

Pladienolide B is a natural macrolide isolated from *Streptomyces platensis* in 2004 as a strong suppressor of hypoxia-induced gene expression [71]. Pladienolide B was shown to arrest cell cycle progression at G1 and G2/M phases and to exert potent anti-tumor activity in human cancer cell lines and mouse xenografts [72]. Subsequent photo-affinity labelling studies identified SF3B1 as the major target of pladenolide B [73] and revealed that the N1074 residue in SF3B1 is required for high binding affinity to the drug [74]. These findings clearly demonstrated that the anti-tumor activity of pladienolide B is exerted through the inhibition of the SF3b complex and splicing (Figure 3).

As described for the FR901464 analogues, crystal structure analyses showed that pladienolide B stalls SF3B1 in an open conformation and affects its transition to the closed conformation that is required to promote the formation of the BPS-U2 snRNA duplex [45]. Nanopore analysis of nascent RNA processing indicated that inhibition of BPS recognition by pladienolide B causes a rapid and global reduction of co-transcriptional splicing [86]. This observation indicates a tight crosstalk between the transcription and splicing machineries, in line with the observation that treatment with pladienolide B, or SSA, decreases the phosphorylation of Serine 2 residues in the carboxyl-terminal domain (CTD) of the RNA Polymerase II (RNAPII) [87], a post-translational modification involved in the coupling of transcription and mRNA processing [88]. Moreover, recent studies showed that inhibition of BPS recognition by pladienolide B increases the duration of RNAPII pausing in the promoter-proximal region and reduces its elongation rate in proximal regions of genes [89]. Thus, by impairing the formation of a functional spliceosome, pladienolide B interrupts the positive feedback between the splicing and transcription machineries that is necessary for an efficient RNA synthesis from human genes.

Importantly, pladienolide B is effective in human cancers for which no therapeutic strategy is currently efficacious. For instance, prostate cancer is dependent on androgen signaling, and anti-androgenic therapy (ADT) is highly efficacious at early stages. However, most prostate cancers evolve to an incurable castration-resistant phenotype. Several mechanisms of ADT resistance have been described, including aberrant splicing of the androgen receptor to yield constitutively active variants [90]. Pladienolide B treatment caused the regression of tumors bearing these androgen receptor variants, indicating that splicing inhibition could be exploited to target prostate cancers that have acquired resistance to standard therapies [91]. Another incurable human cancer is pancreatic ductal adenocarcinoma (PDAC), which displays insensitivity to chemotherapy and a 5-year overall survival <10%. PDAC is driven by mutations in the KRAS and TP53 genes, and these mutations collaborate to promote tumorigenesis by globally altering the splicing program [92]. Importantly, mutant p53 sensitized PDAC tumors to splicing inhibition by two derivatives of pladienolide B, E7107 and H3-8800 (see below). Furthermore, SF3B1 was found to be frequently upregulated in advanced PDAC cases and associated with tumor grade and pladienolide B strongly repressed tumor-related features [93]. These reports pave the ground for investigation of spliceosome inhibitors in the clinical setting of PDAC and hold promise that these compounds may improve the response of this deadly tumor to chemotherapeutic treatments. 

E7107 is a derivative of pladienolide B that also blocks spliceosome assembly and elicits widespread splicing dysregulation [66,73]. E7107 binds in the branchpoint adenosine-binding pocket of SF3B1, near residues that confer resistance upon mutation, such as R1074, suggesting that the drug interferes with BPS recognition in a competitive manner [94]. The promising results obtained in preclinical studies fostered its application in the clinical setting. E7107 was initially tested in a phase I dose-escalation trial (NCT00459823) to assess its safety, tolerability, pharmacokinetics, pharmacodynamics, and clinical activity in patients with solid tumors refractory to standard therapies. The study confirmed the dose-dependent inhibition of pre-mRNA processing by E7107 in vivo, with reversible adverse effects [67]. However, another trial (NCT00499499) revealed that two patients experienced vision loss [68]. More recently, an ex vivo longitudinal observational phase II trial (NCT04555473) was started to evaluate the reliability of high grade serous ovarian carcinoma (HGSOC) organoids as a model for the patients’ response to treatments [95]. The rationale for this study is based on MYC amplification in HGSOC (TCGA Firehouse Legacy), which could possibly expose these tumors to vulnerability to splicing inhibition.

The recently developed H3B-8800 is also derived from pladienolide B and inhibits splicing by targeting SF3B1. H3B-8800 preferentially induces the retention of GC-rich introns in the pre-mRNAs of genes encoding splicing factors. However, with respect to E7107, H3B-8800 is orally available and preferentially targets epithelial and hematologic tumor cells harboring mutations in spliceosome genes [63]. H3B-8800 was shown to synergize with proteasome inhibitors in T-cell acute lymphoblastic leukemia (T-ALL), where the splicing machinery is stabilized and up-regulated through de-ubiquitination of splicing factors [96]. The specificity of H3B-8800 towards cancer cells bearing alterations in the splicing machinery suggests that this compound may spare normal tissue cells in vivo and elicit less adverse effects. On this basis, in 2016, H3B-8800 entered phase I clinical trials enrolling patients with MDS, AML, and CMML (NCT02841540). Initial results revealed dose-dependent target engagement and favorable pharmacokinetics and safety profiles, even with prolonged dosing. Although objective therapeutic responses have not been reported to date, 14% of patients had reduced requirements for red blood cell or platelet transfusion [76]. These data indicate the therapeutic potential of splicing modulation in patients and definitive results are awaited to determine whether H3B-8800 can translate into therapeutic regimens.

### 5.3. GEX1 and Its Derivative Herboxidiene

GEX1 was first isolated from a culture broth of *Streptomyces sp. II.* during a screening for antibiotics displaying anti-tumor activities [97]. GEX1A, the compound with the highest cytotoxic properties, was also reported as an herbicide (named herboxidiene) [98]. Similar to other splicing inhibitors, GEX1 compounds induced both G1 and G2/M arrest in different human tumor cell lines. 

GEX1A/herboxidiene have a quite complex molecular structure that contains nine chiral centers. Several structural derivatives of herboxidiene have been synthesized and evaluated for their splicing activity. Genome wide transcriptome analysis demonstrated that Pladenolide B and GEX1A/herboxidiene display similar effects on gene expression and splicing patterns, indicating that they target the same molecular components [99]. Interestingly, both drugs induce stress signals in plants by activating the abscisic acid (ABA) stress-signaling pathway [100]. The plant hormone ABA plays a crucial role in plant development and responses to abiotic stresses. By modulating alternative splicing of positive and negative regulators of the ABA pathway and activating ABA-induced promoters, Pladenolide B and GEX1A/herboxidiene, mediate ABA response to stress. Remarkably, SSA does not have an effect on plant growth and development, underlying the possibility of a different regulation [75].

Although chemically distinct, all mentioned compounds act by binding to the HEAT repeats domain of SF3B1 and preventing the transition to a closed conformation that recognizes the BPS, which is essential for the first step of catalysis by bringing together the branch-site adenosine and the 5′ splice site (Figure 3) [45]. These splicing modulators arrest spliceosome assembly prior to the ATP-dependent formation of a stable A complex, leading to an “A-like” complex where U2 snRNP is less stably bound and the base pairing interactions between U2 and the intron are altered [99,101]. However, these structurally similar drugs present differential effects, revealing surprising plasticity in the response to splicing modulatory compounds.

### 5.4. Indisulam and Its Derivatives

A different type of spliceosome inhibitors is represented by Indisulam (also known as E7070). Indisulam is a sulfonamide derivative that was initially reported to inhibit carbonic anhydrases and cytosolic malate dehydrogenase [102]. However, later studies demonstrated that the anti-tumor activity of Indisulam relies on the indirect inhibition of RBM39, a U2AF65-like protein involved in the recognition of the 3′ splice site [103,104]. Indisulam acts as a molecular glue to recruit RBM39 to DCAF15 through its RRM2 (Figure 3). DCAF15 is an adapter protein for the CUL4/Ddb1 E3 ubiquitin ligase, and its interaction with RBM39 mediates its ubiquitination at the N terminus and degradation [103,104,105]. Although the paralogue RBM23 protein is also degraded through the same DCAF15-mediated mechanism, the widespread gene expression and splicing dysregulation exerted by Indisulam in human cells could be entirely attributed to RBM39 [105]. The strict dependency of Indisulam action on DCAF15 suggests that this splicing inhibitor might be particularly effective in cells expressing high levels of this protein or acquired dependency on RBM39 functions. For instance, DCAF15 is upregulated in some acute myeloid leukemia (AML) patients with respect to normal hematopoietic progenitors and AML cells which showed strong dependency on RBM39 expression [106]. Indisulam strongly repressed RBM39 expression and viability of AML cells in vitro and in vivo, while sparing normal hematopoietic cells. Moreover, both Indisulam and its derivative E7820 were highly effective on AML cells harboring mutations in splicing factors known to interact with RBM39, such as SF3B1 and U2AF35 [106]. Thus, mutations in spliceosomal genes and/or high DCAF15 expression could be used to predict response to Indisulam and help select patients that might benefit from this treatment, which demonstrated an excellent safety profile in clinical trials. These findings may also explain why Indisulam and related compounds exhibited low overall response rates in clinical trials in which patients were not rationally selected on the basis of their mechanism of action (i.e., NCT00003981). A more recent phase II trial (NCT01692197) in patients with relapsed and/or refractory AML or high-risk MDS revealed the beneficial effects of Indisulam administration in combination with chemotherapy, with an estimated 1-year overall survival of 51% in responders compared with 8% in non-responders [77]. It is likely that selection of AML patients for DCAF15 expression and/or spliceosome mutations will further improve the clinical efficacy of Indisulam and its derivatives.

Another feature that confers susceptibility to Indisulam is amplification or overexpression of the oncogene MYC. As described above, MYC-driven cancers were already known to be particularly vulnerable to splicing inhibition by SF3B-targeting compounds [57]. However, a recent study highlighted that MYC-driven neuroblastoma strictly depends on RBM39 expression for survival. Indisulam treatment was very efficacious in neuroblastoma disease models, due to their dependency on RBM39 and the high-level of DCAF15 expression [107]. These observations further confirm that monitoring DCAF15 expression can predict sensitivity to splicing inhibition by this drug.

In addition to directly targeting cancer cell survival, Indisulam was recently shown to enhance immunotherapeutic strategies for cancer treatment. Indeed, at suboptimal doses, Indisulam did not affect cancer cell viability, but it altered splicing and yielded immunologically valuable neoepitopes that elicited anti-tumor immunity and enhanced the efficacy of checkpoint immunotherapy [108]. At these doses, Indisulam blocked tumor cell growth only in vivo and in a host T cell-dependent manner. Moreover, by combining bioinformatics analyses with functional biological validation experiments, it was shown that Indisulam favored the production of peptides that were efficiently presented by the major histocompatibility complex class I (MHC-I). Notably, similar results were also observed with a mechanistically distinct splicing inhibitor [108], which targets the protein arginine methyltransferase 1 (PRMT1; see next section). Collectively, this elegant and thorough study demonstrates that splicing modulation by chemical drugs can generate immunogenic neoepitopes “on demand”, thus also potentially rescuing sensitivity to immune checkpoint blockade in the so-called “cold” tumors. Since Indisulam proved to be well tolerated in vivo (NCT00003981), these results have the potential to be readily translated to the clinic.

### 5.5. Inhibition of the Protein Arginine Methyltransferase PRMT1 and PRMT5

Arginine methylation is a common post-translational modification implicated in many biological processes such as transcription, splicing, and signal transduction [109,110]. Splicing factors and RBPs are highly enriched in glycine-arginine rich (GAR or RG/RGG) motifs and Tudor domains, which are both targeted by PRMTs [111]. For example, small nuclear ribonucleoproteins (snRNPs) are assembled in the cytoplasm through interactions between uridylate-rich small nuclear RNAs (U-snRNAs) and the Sm proteins SmB/B′, SmD1, and SmD3 (Figure 3). PRMT5-mediated arginine methylation of the GAR motifs in the C-terminal domain of Sm proteins increases the hydrophobicity of the arginine side chain and favors its interaction with aromatic cages, thus altering protein–protein and protein–RNA interactions [112,113,114]. As a consequence, deletion or inhibition of PRMT5 results in aberrant splicing [115]. Treatment of mantel cell lymphoma (MCL) cell lines with the orally available PRMT5 inhibitor EPZ015666 (GSK3235025) led to inhibition of SmD3 methylation and cell death, while oral administration in MCL xenograft mouse models demonstrated dose-dependent anti-tumor activity [116]. Accordingly, treatment of human cancer cell lines with the EPZ015666 derivatives GSK3203591 and GSK3326595 impacts cell growth by inducing defects in mRNA splicing [78].

Glioblastoma multiforme (GBM) is an aggressive cancer for which no cure is currently available. This tumor type appears particularly vulnerable to splicing dysregulation elicited by PRMT5 inhibition or knockdown. EPZ015666 was shown to suppress GBM growth in vivo by disrupting splicing of introns enriched in genes involved in cell proliferation, thus causing cell cycle arrest and apoptosis [117]. These results were confirmed by a subsequent study that employed two different PRMT5 inhibitors (GSK591 and LLY-283). By eliciting widespread splicing defects, these inhibitors inhibited the growth of GBM stem cells, with the pro-neural subtype showing the greatest sensitivity [118]. Notably, LLY-283 was able to pass the blood–brain barrier, suggesting that it might serve as template for the development of clinically valuable drugs for GBM treatment.

As for other splicing inhibitors, the mutational status of cancers can enhance susceptibility to PRMT5 inhibitors. For instance, cells harboring deletion of the methyl-thioadenosine phosphorylase (MTAP) gene are particularly sensitive to inhibition of the metabolic enzyme methionine adenosyltransferase 2α (MAT2A) [119]. MTAP and MAT2A contribute to maintain the cellular levels of S-adenosylmethionine (SAM), the substrate used by PRMT5 and other PRMTs for their catalytic activity. MAT2A inhibition in MTAP-deleted cells leads to reduced PRMT5 activity and widespread splicing defects [119]. These experiments suggest that, in this genetic context, MAT2A inhibition may lower the effective threshold of PRMT5 inhibitors. Furthermore, another study revealed that MTAP deletion results in accumulation of the metabolite 2-methylthioadenosine and promotes the synergism between inhibitors of PRMT5 and of type I PRMT (i.e., GSK3368715) in PDAC [79], a tumor type in which MTAP is frequently deleted. The safety and efficacy of GSK3368715 and association of its activity with the MTAP status in patients is currently being evaluated in a clinical trial (NCT03666988).

PRMT5 catalyzes symmetric dimethylation of the terminal amino group of the arginine side chain, whereas PRMT1, the most abundant of type I PRMTs, catalyzes asymmetric demethylation [111]. The synergism observed with inhibitors of these enzymes are consistent, with each of them having distinct substrates [79]. Indeed, while PRMT5 preferentially modifies Sm proteins, PRMT1 modifies several RBPs, including the splicing regulator RBM15 [120] and the chromatin target of PRMT1 (CHTOP) protein [121]. PRMT1-mediated methylation of RBM15 at residue R578 caused its ubiquitination and degradation and impaired alternative splicing by altering SF3B1 recruitment to specific introns [120]. Moreover, class I PRMT inhibitors have been recently shown to modulate widespread post-transcriptional splicing of many introns through direct modulation of CHTOP methylation. Notably, PRMT inhibitors impaired splicing fidelity and elicited higher antiproliferative activity toward splicing factor-mutated leukemias over wild-type counterparts [122], further providing support for therapeutic strategies based on the presence of specific genetic alterations. In addition, a functional genomics screening using patient-derived PDAC models has recently identified PRMT1 as a suitable target for inhibition of PDAC growth. PRMT1 inhibition globally altered splicing, poly-adenylation, and transcription termination, ultimately leading to uncontrolled genomic instability [123].

Of note, PRMT inhibitors were highly effective against currently incurable cancers that display an extremely poor prognosis, such as GBM and PDAC [117,118,123]. This observation raised considerable clinical interest on the possibility to target splicing through inhibition of PRMTs. Inhibitors of PRMT5 and PRMT1 are in phase I clinical trials for patients with advanced-stage hematological malignancies (NCT02783300, NCT03886831) and solid tumors (NCT04794699), including breast (NCT04676516) and colorectal cancers (NCT02022995). Moreover, a phase II trial aims at testing these inhibitors on HGSOC patient-derived organoids (NCT04555473). Results from these trials will shed light on the clinical efficacy of these inhibitors and may help translate splicing inhibition into treatments for several human cancers.

### 5.6. UHMCP1 and the Inhibition of SF3B1/U2AF65 Interaction

Interactions between the U2AF homology motifs (UHMs) and U2AF ligand motifs (ULMs) play a crucial role in early spliceosome assembly. UHM–ULM interactions mediate heterodimerization of the constitutive splicing factors U2AF65 and U2AF35 and between other splicing factors that regulate spliceosome assembly at the 3′ splice site [124]. Recently, a high throughput screening identified a small molecule, UHMCP1, which prevents the SF3B1/U2AF65 interaction. UHMCP1 interacts with the hydrophobic pocket of the U2AF65 UHM domain, thus strongly impacting RNA splicing and cell viability of cancer cells [80]. Another study identified phenothiazine derivatives, such as 7,8-dihydroxyperphenazine and 7,8-dimethoxyperphenazine, as molecules targeting the early assembly of the spliceosome [81]. In particular, the 7,8-dimethoxyperphenazine inhibits the formation of complex A by inhibiting UHM–ULM interactions between U2AF65 and SF1 and between U2AF35 and U2AF65 and, during the transition from E to A complex, by inhibiting UHM–ULM interactions between U2AF65 and SF3B1 [81]. Furthermore, the 7,8-dihydroxyperphenazine inhibitor blocks the formation of the B complex, by inhibiting the binding of U4/U6-U5 tri-snRNP to the A complex [81]. Beyond U2AF2, these compounds inhibit other UHM domain-containing proteins, including RBM39, SPF45, and PUF60 [81]. Further work is needed to optimize the in vivo potency of these compounds and to define their therapeutic potential.

## 6. Concluding Remarks

In this review, we have discussed the relevance and therapeutic implications of recurrent somatic mutations in genes encoding spliceosomal components in human cancers. Notably, the identified mutations predominantly occur in hematological malignancies, highlighting the possibility of a selective tissue-specificity. It is possible that these heterozygous mutations are not sufficient to induce cell transformation in other tissues, while being pernicious only to hematopoietic stem cells, eventually in combination with other oncogenic events [32]. According to this hypothesis, alterations in the splicing process may initially infringe cell homeostasis by producing aberrant protein isoforms critical for disease pathogenesis, which, in the long-term, lead to the accumulation of compensatory adaptive changes driving neoplastic transformation and cancer progression. On the other hand, cloning analyses from the bone marrow of subjects with increasing age revealed gradually accumulating changes in a linear fashion from birth to the adulthood, driven by independent mutational processes [125]. Thus, the mutational signature of hematopoietic stem cells could only reflect an age-dependent but constantly active process during life, which leads to the characteristic clonal accumulation, instead of a specific tissue vulnerability.

Herein, we have also described small molecules that globally perturb RNA splicing and are currently being tested in clinical trials for the treatment of cancers, especially those harboring splicing factor mutations. Importantly, these molecules target an essential function in the cell, such as the processing of introns from pre-mRNAs. Thus, a complete block of splicing would result in the lethality of all cells, not only cancer cells. Nevertheless, most of these drugs alter the fidelity and efficiency of splicing, without completely blocking the process. Moreover, some tumors appear to be significantly more sensitive to these drugs than normal cells of the same tissues. This increased vulnerability may rely on the higher dependency on splicing of cancer cells with increased transcriptional activity, such as MYC-driven tumors [57] and tumors displaying addiction to oncogenic transcription factors, such as the AR in prostate cancer [90,91]. Thus, future efforts should also be directed at stratification of tumor types and/or patients that may benefit from splicing–targeting approaches based on predictive molecular signatures. This approach may allow for lowering the doses of these drugs to concentrations that are sub-optimal for normal cells while being efficacious for cancer cells.

The discovery of key pathologic RNA splicing changes generated by cancer-driving mutations in splicing factors has also highlighted vulnerabilities in these genetic subtypes of cancer that can be exploited therapeutically by developing splice-switch antisense oligonucleotides (ASOs). To date, three ASO therapies targeting specific splicing events have been approved by the FDA, Nusinersen (Spinraza) for the treatment of spinal muscular atrophy (SMA), etepliresen (Exondys 51), and golodirsen (Vyondys 53) for the treatment of Duchenne muscular dystrophy (DMD) [126]. Similar strategies could be pursued to correct recurrent splicing abnormalities in specific cancer subtypes. The combination of small molecule inhibitors with ASO technologies could be instrumental for the development of more effective and targeted therapies.

## Figures and Tables

**Table 1:** Small molecules inhibiting RNA splicing in cancer.

Drug	Target	Short Description	Clinical Trials	Reference
FR901464	SF3b	Anti-tumor activity in lung, breast cancer, and other cancers	-	[64,65]
E7107	SF3b	Block spliceosome assembly in patients with solid tumors	NCT00459823 NCT00499499 NCT04555473 NCT03729453	[66,67,68]
Meayamycin	SF3b	Induction of apoptosis in head and neck cancer cells	-	[69]
Sudemycin	SF3b	Induce anti-tumor response in chronic lymphocytic leukemia	-	[65,70]
Pladienolides	SF3b	Display anti-proliferative effects	-	[71,72,73,74]
GEX1A	SF3b	Anti-tumor activity by targeting SF3B1 protein		[75]
H3B-8800	SF3b	Anti-tumor activity by targeting SF3B1 protein	NCT02841540	[63,76]
Indisulam	RBM39	RBM39 degradation in the hematopoietic and lymphoid tissues	NCT00165867 NCT00014625 NCT00003981 NCT00003976 NCT00165594 NCT00165880 NCT01692197 NCT00059735	[77]
EPZ015666	PRMT5	Inhibition of PRMT5 enzymatic activity	-	[78,79]
UHMCP1	U2AF65	Inhibitor of SF3B1/U2AF65 interaction	-	[80,81]
